# Development of coronary dysfunction in adult progeny after maternal engineered nanomaterial inhalation during gestation

**DOI:** 10.1038/s41598-021-98818-8

**Published:** 2021-09-29

**Authors:** Sara B. Fournier, Vincent Lam, Michael J. Goedken, Laura Fabris, Phoebe A. Stapleton

**Affiliations:** 1 Environmental and Occupational Health Sciences Institute, Rutgers University, 170 Frelinghuysen Road, Piscataway, NJ 08854 USA; 2 Department of Pharmacology and Toxicology, Ernest Mario School of Pharmacy, Rutgers University, 160 Frelinghuysen Rd., Piscataway, NJ 08854 USA; 3 Research Pathology Services, Rutgers University, 41 Gordon Road, Piscataway, NJ 08854 USA; 4 Department of Material Science and Engineering, School of Engineering, Rutgers University, 607 Taylor Rd., Piscataway, NJ 08854 USA

**Keywords:** Nanotoxicology, Cardiovascular biology, Reproductive biology

## Abstract

Maternal exposure to environmental contaminants during pregnancy can profoundly influence the risk of developing cardiovascular disease in adult offspring. Our previous studies have demonstrated impaired cardiovascular health, microvascular reactivity, and cardiac function in fetal and young adult progeny after maternal inhalation of nano-sized titanium dioxide (nano-TiO_2_) aerosols during gestation. The present study was designed to evaluate the development of cardiovascular and metabolic diseases later in adulthood. Pregnant Sprague–Dawley rats were exposed to nano-TiO_2_ aerosols (~ 10 mg/m^3^, 134 nm median diameter) for 4 h per day, 5 days per week, beginning on gestational day (GD) 4 and ending on GD 19. Progeny were delivered in-house. Body weight was recorded weekly after birth. After 47 weeks, the body weight of exposed progeny was 9.4% greater compared with controls. Heart weight, mean arterial pressure, and plasma biomarkers of inflammation, dyslipidemia, and glycemic control were recorded at 3, 9 and 12 months of age, with no significant adaptations. While no clinical risk factors (i.e., hypertension, dyslipidemia, or systemic inflammation) emerged pertaining to the development of cardiovascular disease, we identified impaired endothelium-dependent and -independent arteriolar dysfunction and cardiac morphological alterations consistent with myocardial inflammation, degeneration, and necrosis in exposed progeny at 12 months. In conclusion, maternal inhalation of nano-TiO_2_ aerosols during gestation may promote the development of coronary disease in adult offspring.

## Introduction

Early life exposures profoundly influence the risk of developing disease later in life. This concept, identified as the Developmental Origins of Health and Disease (DOHaD) paradigm, hypothesizes that environmental exposures during critical periods of growth and development in early life (i.e. gestation) may predispose an individual to chronic disease in adulthood. The most notable examples describe the development of cardiovascular and metabolic diseases in offspring, by Barker^1^. In these seminal studies, Barker and colleagues present the intrauterine origins of cardiovascular and metabolic disease and identify strong correlations between intrauterine or fetal growth restriction and the development of cardiovascular and metabolic diseases later in life^2–4^.

More recent studies demonstrate maternal exposure to environmental contaminants results in increased risk of adult disease in offspring^5^. Unfortunately, of the 425 publications identified between 1988 and 2014, only 15% focused on cardiovascular or metabolic outcomes; furthermore, only 28 publications focused on in utero exposures only and none evaluated air pollution or particulate matter (PM) exposure as an initiating source^5^. Epidemiological evidence demonstrates that exposure to ambient particulate matter, at any point between pre-conception and birth, results in reduced fetal growth^6^. Additionally, data derived from the Boston Birth Cohort suggests that maternal exposure to fine ambient PM (PM_2.5_) during the third trimester is associated with increased blood pressure in children^7^. Ultimately, exposure to air pollution early in life has been linked to the development of cardiovascular disease^8^ and impaired cardiac function^9,10^. The progression of cardiovascular disease has been attributed to increased risk factors, including obesity, hypertension and metabolic disease^8^. In laboratory mouse models, in utero exposure to diesel exhaust PM results in increased weight gain, reduced blood pressure, and cardiac hypertrophy attributed to pressure overload culminating in an increased susceptibility to heart failure in male offspring at 3 months of age^11,12^. Interestingly, male mice directly exposed to diesel exhaust PM did not exhibit symptoms of heart failure for up to 6 months post-exposure^13^, suggesting that these outcomes are directly associated with gestational exposure.

Engineered nanomaterials (ENM) have been previously utilized as a surrogate to represent ultrafine PM (PM_0.1_)^14^. Presently, titanium dioxide nanomaterials (nano-TiO_2_) are one of the most prolific nanoparticles applied to domestic and industrial products. The production of nano-TiO_2_ has increased substantially over the last decade, with an estimated 2.5 million tons to be produced in 2025^15^. Studies focused on occupational exposure, regulation and risk management of nano-TiO_2_ have identified associations between occupational exposure to nano-TiO_2_ and pulmonary inflammation, oxidative stress, cytotoxicity, and fibrosis^16,17^. Based on experimental evidence from inhalation studies, TiO_2_ has been classified by the International Agency for Research on Cancer (IARC) as possibly carcinogenic to humans^18^.

Studies of maternal exposure to nano-TiO_2_ during gestation has been shown to impair maternal and offspring health^19–24^. Unfortunately, data focused on the development of cardiovascular and metabolic disease after in utero exposure to nano-TiO_2_ is limited^19,25^. Previous studies have identified plausible mechanisms of progeny impairment following maternal exposure to TiO_2_ during pregnancy including fetal growth restriction, particle translocation, systemic inflammation, oxidative stress, and epigenetic modifications^19,25–27^. These conditions may promote the development of traditional cardiovascular risk factors including hypertension, obesity, dyslipidemia, and diabetes. We identified a 32% reduction in fetal growth in litters from dams exposed to nano-TiO_2_ aerosols via whole-body inhalation for more than 10 days of pregnancy^24^. Evaluations of female progeny have identified impaired endothelium-dependent dilation of the coronary microvasculature at 3 months; additionally, there have been significant reductions in mitochondrial respiration in the left ventricle providing evidence of an association between cardiovascular and metabolic impairments and gestational exposure to nano-TiO_2_^28^. Further, a study by Hathaway et al. reported functional impairments to cardiomyocyte function and bioenergetics in young adult rats after maternal exposure to nano-TiO_2_ aerosols throughout gestation^22^.

While these initial studies have demonstrated impairments in cardiovascular health, microvascular reactivity and cardiac function in young adult progeny after maternal exposure to nano-TiO_2_ aerosols during gestation, the timeline of disease development and progression remains unclear. Furthermore, traditional clinical markers of cardiovascular and metabolic disease have not yet been evaluated. Therefore, the purpose of this study was to track traditional markers of cardiovascular and metabolic disease in progeny from birth to 1 year of age after maternal inhalation of nano-TiO_2_ particles during gestation. Our results provide evidence that gestational exposure to nano-TiO_2_ significantly impairs progeny coronary health; however, this outcome is not associated with the presentation of traditional cardiovascular risk factors including obesity, dyslipidemia, systemic inflammation, or impaired glycemic control.

## Results

### Litter characteristics

Average length of gestation, average number of pups per litter, and sex ratio (expressed as percent male) are reported in Table 1. No differences in litter characteristics were observed between sham-control and exposure groups.

**Table 1:** Litter characteristics.

Group	Number of litters (n)	Gestation length (days)	Average pups per litter	Male/female (%)	Mortality	Health concerns
AIR	4	22.25 ± 0.25	11.00 ± 0.41 (n = 11)	50.00 50.00	3	Tumor (1)
ENM	4	22.50 ± 0.50	12.75 ± 0.48 (n = 12–14)	56.00 44.00	2	Seizure (1)

Values are shown as mean ± SEM. Statistics were analyzed with a students T-test (*p* < 0.05).

### Progeny growth

**Figure 1:**
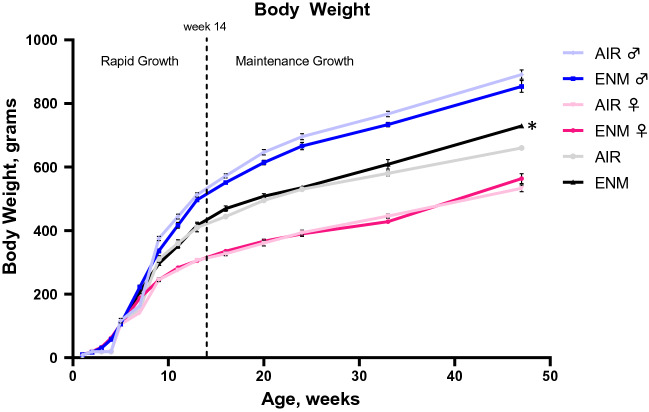
Body weight. Interval body weight (mean ± SEM) versus Age (weeks) of offspring from Sham-Control and ENM groups. *Indicates *p* ≤ 0.05 versus Sham-Control (AIR) group.

Average interval body weights of offspring until 1 year of age are presented in Fig. 1. The average body weight of nano-TiO_2_ exposed offspring are similar compared with average body weight of offspring exposed to filtered air in the first 3 months (rapid growth phase). The average body weight of nano-TiO_2_ exposed offspring was significantly higher compared with the average body weights of sham-control offspring in the 15-week moving average reported at week 47 (Fig. 1) (*p* < 0.01, two-way ANOVA followed by LSD post hoc multiple comparison). Average body weights of ENM-exposed offspring tended to be greater compared with average body weights of sham-controls during the maintenance phase of growth, however these differences were not significant. At week 47, average body weight from offspring exposed to nano-TiO_2_ was approximately 9.4% greater compared with average body weight of offspring exposed to filtered air.

### Progeny blood pressure and heart weight

**Figure 2:**
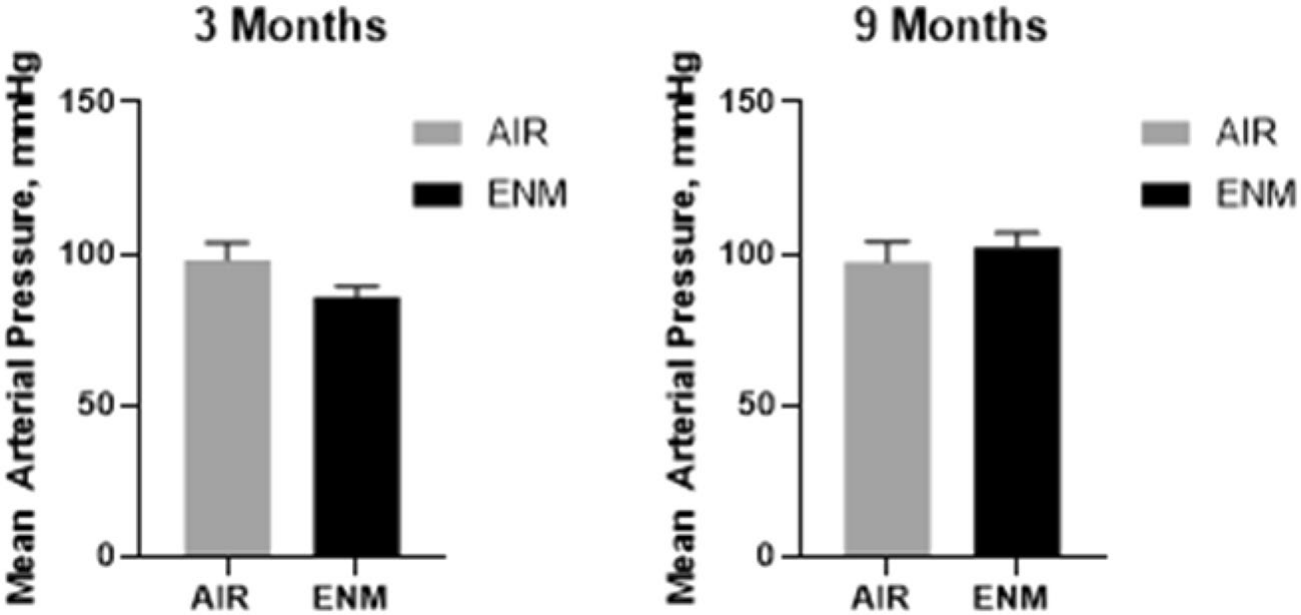
Mean Arterial Pressure. Mean arterial pressure (MAP) for Sham-Control (gray bars) and ENM (black bars) offspring at 3 and 9 months of age. Values are shown as mean ± SEM.

**Figure 3:**
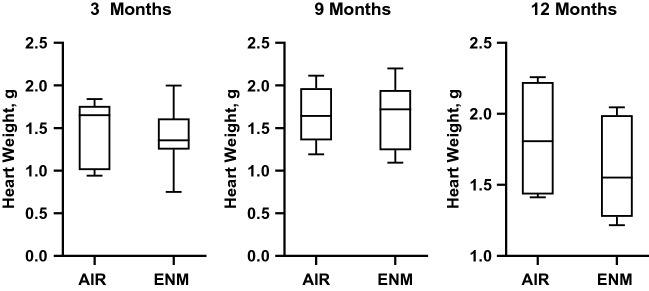
Heart Weight. Heart weight of offspring in Sham-Control (gray bars) and ENM (black bars) offspring at 3, 9, and 12 months of age. Values are shown as mean ± SEM. Statistics were analyzed with a Students T-test (*p* < 0.05).

There were no significant differences in MAP (Fig. 2) or heart weight (Fig. 3) in exposed offspring compared with control offspring at 3, 9 or 12 months. This suggests the offspring exposed to nano-TiO_2_ aerosols during gestation do not develop systemic hypertension or cardiac hypertrophy.

### Coronary vascular reactivity

At 12 months, pressure myography was used to evaluate microvascular reactivity of isolated coronary arterioles in response to chemical stimuli. The characteristics of these arterioles can be found in Table 2. Inner diameter and wall thickness were recorded after vessel equilibration and prior to the addition of any chemical agonists; the passive measures were recorded after the reactivity assessments and the addition of Ca^2+^-free PSS (Table 2). No significant differences between the control and exposed arterioles were noted.

**Table 2:** Coronary arteriole characteristics.

Group	n	Inner diameter (µm)	Wall thickness (µm)	Tone (%)	Passive outer diameter (µm)	Passive wall thickness (µm)
AIR	4	67.50 ± 16.48	25.13 ± 1.59	48.40 ± 12.36	183.50 ± 27.50	23.50 ± 1.68
ENM	5	96.00 ± 16.54	26.40 ± 3.21	40.25 ± 11.96	211.20 ± 9.22	22.00 ± 3.52

Coronary arteriole characteristics in Sham-control and ENM groups. Values are shown as mean ± SEM. Statistics were analyzed with a students T-test (*p* < 0.05).

**Figure 4:**
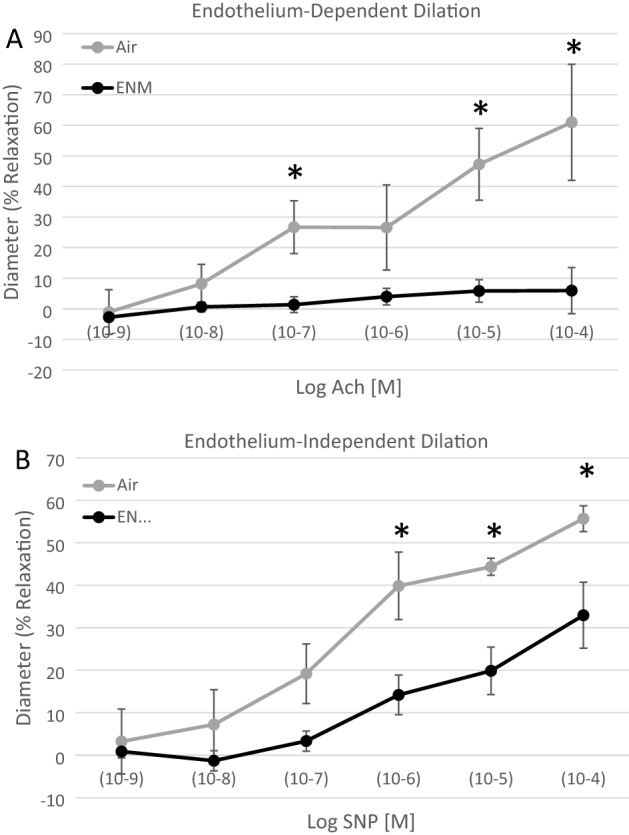
Vascular reactivity of coronary resistance arterioles at 12 months. (**A**) Endothelium-dependent dilation of coronary arterioles from Sham-Control and nano-TiO_2_-exposed animals at 12 months was determined using pressure myography. ACh, Acetylcholine. (**B**) Endothelium-independent dilation of coronary arterioles from Sham-Control and nano-TiO_2_-exposed animals at 12 months was determined using pressure myography. SNP, Sodium nitroprusside. Values are shown as mean ± SEM. n = 4–5. Statistics were analysed with two-way ANOVA., *Indicates *p* ≤ 0.05 versus Sham-Control (AIR) group.

To assess endothelium-dependent [acetylcholine (ACH, 1 × 10^−9^ to 1 × 10^−4^ M)], and endothelium-independent [sodium nitroprusside (SNP, 1 × 10^−9^ to 1 × 10^−4^ M)], reactivity. Endothelium-dependent (Fig. 4A) and -independent (Fig. 4B) reactivity was significantly blunted in exposed offspring compared with controls.

### Histopathology

**Figure 5:**
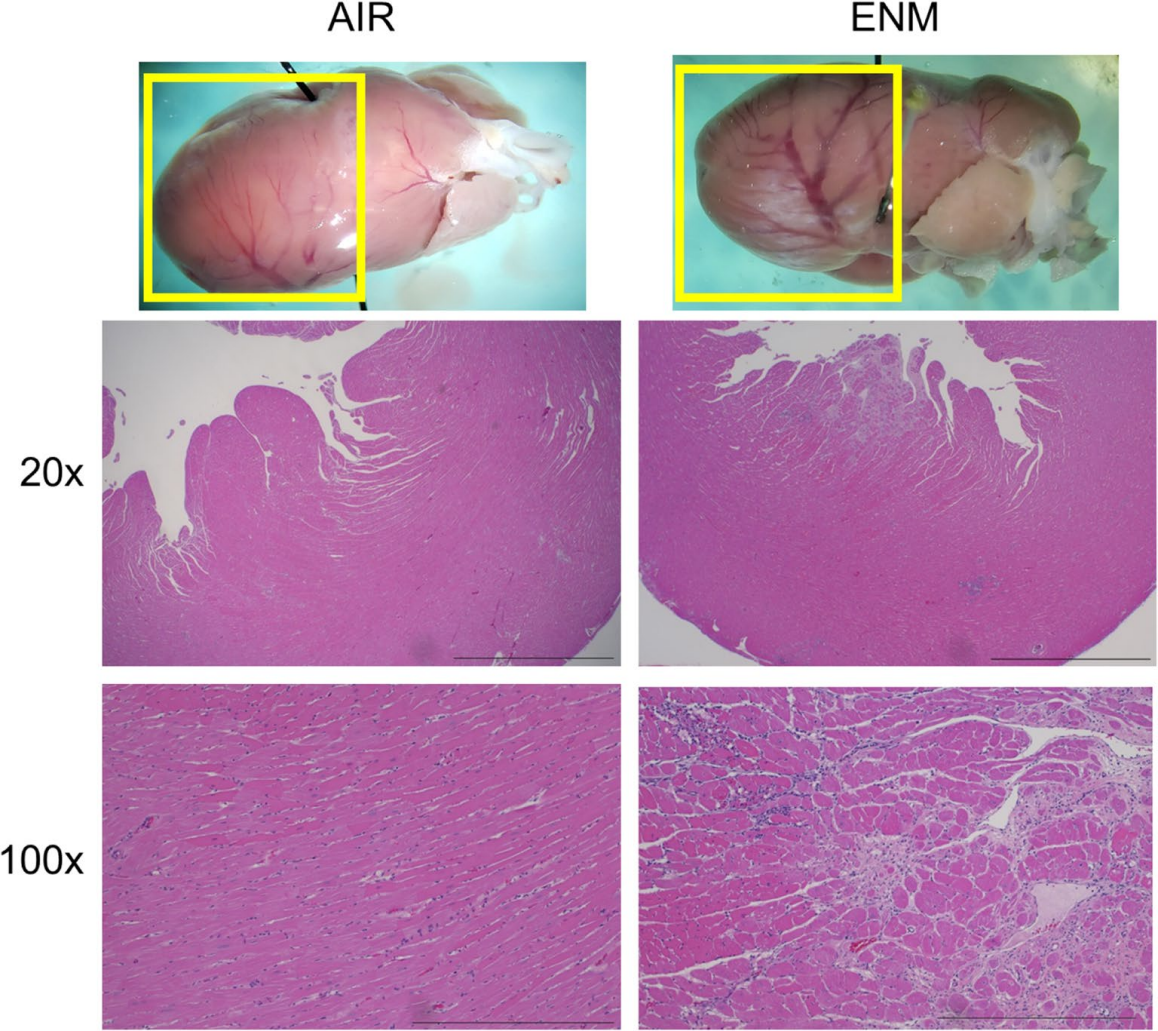
Histological analysis of cardiac tissue at 12 months. Histological analysis of cardiac tissue from offspring of pregnant dams exposed to nano-TiO_2_ at 12 months revealed histopathological alterations compared with tissue from the Sham-Control (AIR) group. n = 3. Reference bar at 20× = 2 mm. Reference bar at 100× = 500 μm.

Histological analysis of cardiac tissue (Fig. 5) from F1 offspring of pregnant dams at 12 months exposed to nano-TiO_2_ revealed histopathological alterations compared with control tissue. These alterations were characterized by multifocal myocardial inflammation, degeneration, necrosis, loss and/or fibrosis. Findings were principally located in left ventricular free wall subepicardial and/or subendocardial parenchyma. Collectively, these histopathological alterations suggest that chronic in utero exposure to nano-TiO_2_ beginning at GD 4 induces alterations in cardiac morphology that persist into middle age.

### Assessment of metabolic plasma proteins

**Figure 6:**
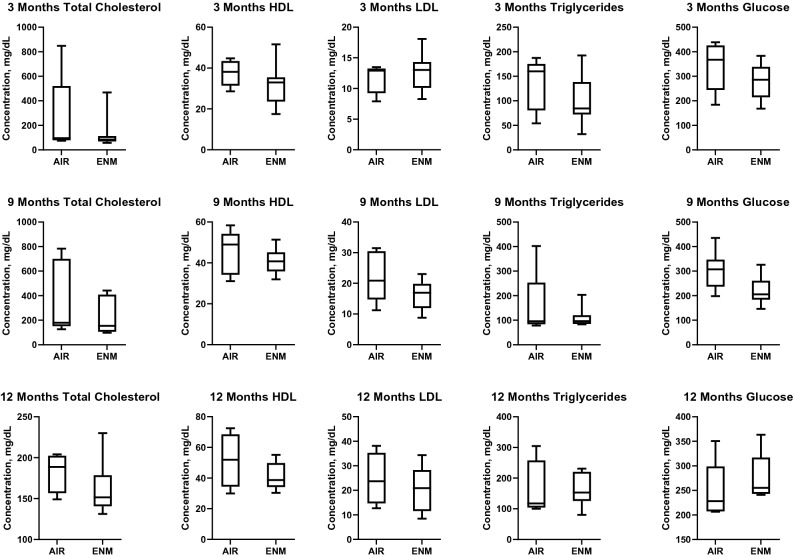
Metabolic panel. Plasma cholesterol, HDL, LDL, and triglycerides were assessed in offspring between Sham-Control or ENM groups at 3, 9, and 12 months of age. HDL, High density lipoprotein; LDL, low density lipoprotein. Values are shown as mean ± SEM.

A lack of significant differences between the groups indicates that chronic exposure to nano-TiO_2_ in utero beginning at GD 4 is not modulating plasma concentrations of cholesterol, HDL, LDL, or triglycerides (Fig. 6).

### Assessment of inflammatory or vascular injury plasma proteins

**Figure 7:**
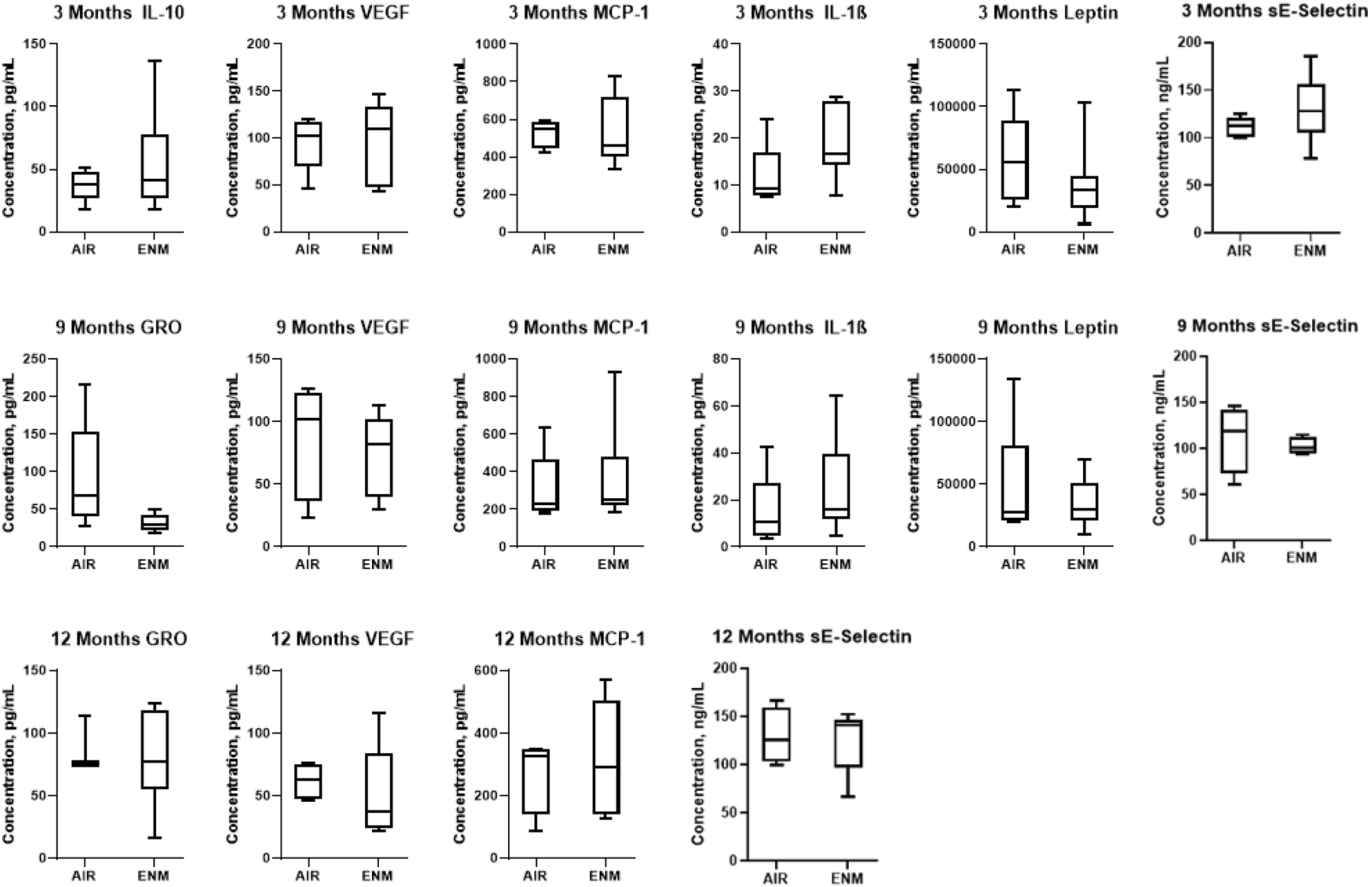
Inflammation and Vascular Injury panel. Plasma IL-10, IL1-β, Leptin, MCP-1, VEGF, GRO, and sE-Selectin were assessed between Sham-Control or ENM offspring at 3, 9, and 12 months of age. IL-10, interleukin 10; IL-1β, interleukin 1 beta; MCP-1, monocyte chemoattractant protein-1; VEGF, Vascular endothelial growth factor. Values are shown as mean ± SEM.

There were no significant differences in the inflammatory markers measured between the control and exposed groups indicating that chronic exposure to nano-TiO_2_ in utero beginning at GD 4 is not modulating plasma concentrations of general inflammatory or vascular injury biomarkers (Fig. 7).

## Discussion

Our studies are the first to evaluate progeny health and the development of cardiovascular disease up to 12 months of age. These results suggest that gestational exposure to nano-TiO_2_ contributes to the development of cardiovascular and coronary heart disease, as presented in Figs. 4 and 5, but this outcome is not associated with the development of traditional cardiovascular risk factors. In this study, animals exposed during gestation weighed more than controls at 47 weeks of age (Fig. 1). Upon further review this outcome was likely driven by an unequal sex ratio between the groups, with more males in the exposed group and more females in the control group, as males are naturally larger than females. While there were 93 progeny in both the control and exposed groups, this outcome was determined by a greater percentage of large males in the exposed cohort (57% male with an average weight of 854 g) and a greater percentage of smaller females (65% female with an average weight of 533 g) in the control group. While the exposed males weighted less than the control males at this time point, shift in the sex ratio likely skewed the combined weight results (Fig. 1).

Exposed offspring presented blood lipid and cholesterol levels that were similar to the control (Fig. 6). Furthermore, there were no systemic markers of inflammation altered during adulthood after gestational exposure to nano-TiO_2_ aerosols (Fig. 7). Therefore, assessments of hypertension, dyslipidemia, glycemia, and inflammation in offspring exposed to nano-TiO_2_ provided null results; yet evaluations of epicardial arteriolar function at 3 months^28^ and 12 months demonstrated endothelium-dependent and -independent dysfunction (Fig. 4).

Endothelium-dependent dysfunction is often associated with a reduction in nitric oxide (NO) bioavailability during the development of cardiovascular disease; however, animals in this study also demonstrated an attenuated endothelium-independent response in the presence of a NO donor (Fig. 4B). These results indicate dysfunction of the relaxation of vascular smooth muscle (VSM) of the epicardial arterioles. This is a unique outcome, and the molecular mechanism(s) of this dysfunction are unclear but may be associated with inhibition of normal VSM, cGMP or NADPH oxidase signalling^29^. Chronic impairment in left ventricular coronary blood flow throughout adulthood may produce the cardiac morphological changes described in Fig. 5. Interestingly, the inflammation and necrosis in hearts from exposed offspring did not lead to an increase in gross heart weight (Fig. 3). These results suggest that the microvascular and coronary impairments observed in this study may not lead to traditional risk factors of cardiovascular disease.

The mechanisms promoting the developmental onset of coronary and cardiac dysfunction after maternal exposure to nano-TiO_2_ aerosols is likely a local multifactorial cascade involving particle translocation, oxidative stress, and epigenetic modifications. Engineered nanomaterials have been identified in the placenta and fetal compartment after within 24-h after maternal pulmonary exposure^20,30^. Recently, studies quantified titanium in human placental and fetal meconium confirming maternal exposure and fetal translocation during pregnancy^31^. Nano-sized materials, including nano-TiO_2_ particles, have been shown to access and accumulate in the fetal heart after maternal exposure^32^. Direct nano-TiO_2_ particle-cellular interactions have been shown to impact human DNA methylation^33^, thus promoting the theory that nano-TiO_2_ translocation to the fetal heart may promote epigenetic mechanisms of cardiac dysfunction. Furthermore, direct nano-TiO_2_ particle and cardiomyocyte interactions reduce cellular metabolic activity and increase oxidative stress^34^. Oxidative stress within BEAS-2B cells has been shown to impact epigenetic processes through increased histone acetylation and decreased methylation^35^. Oxidative stress may also play a role in endothelium-dependent dysfunction and reduced coronary microvascular dilation, further promoting dysregulation of local oxygen delivery and utilization^28^. Therefore increased oxidative stress due to nanoparticle translocation and accumulation in the heart may further alter coronary function.

Due to the transgenerational coronary toxicity, genetic modifications may play a significant role in disease progression after gestational nano-TiO_2_ exposure^19^. Transcriptomic analyses of fetal rat coronary tissue have revealed epigenetic modifications in cardiac, immune, hepatic, renal, and growth signalling^21^, with primary focus on inflammatory signalling and the cardio-hepatic-renal axis. Extrapolation of these alterations into adulthood could manifest as systemic inflammation and impaired blood pressure control; however, neither were reported in the current study. Further study of epigenetic adaptations to offspring after maternal nano-TiO_2_ inhalation during pregnancy identify increased DNA methylation in fetal hearts (GD 15), but this outcome is not sustained in young adult mice at 11 weeks of age^36^. These results may indicate that these epigenetic alterations are not sustained into adulthood, or that physiological compensatory mechanisms may impact functional outcomes. Unfortunately, these studies have yet to be conducted.

Overall, these results identified the development of coronary artery disease and cardiac morphological changes in rats at 1 year of age after gestational exposure to nano-TiO_2_ particles through maternal whole-body inhalation. These cardiovascular perturbations were not attributed to the development of traditional risk factors of cardiovascular disease including obesity, hypertension, dyslipidemia, or impaired glycemic control. These data are vital to the understanding of the DOHaD hypothesis as it pertains to cardiovascular disease and contribute to the concept that maternal environmental exposure during pregnancy may be identified as a risk factor for the development of cardiovascular disease in offspring.

## Materials and methods

### Nanomaterial characterization

Nano-titanium dioxide (nano-TiO_2_) powder was acquired from Evonik (Aeroxide TiO_2_, Parsippany, NJ). Previous characterization of this powder has determined the composition to be anatase (80%) and rutile (20%) TiO_2_, with a primary particle size of 21 nm and a surface area of 48.08 mg^2^/g^24^. Characterization of these particles was re-confirmed via dynamic light scattering (DLS) techniques using Zetasizer Nano ZS by Malvern. The size of the agglomerated nanoparticles in diH_2_O was measured as 231 ± 21 nm with Non-Invasive Backscatter optics (NIBS) using a 4 mW, 633 nm laser^37^. Nano-TiO_2_ powder was dried, sieved, and stored under vacuum prior to aerosolization.

### Experimental animal model

Pregnant, gestational day (GD) 3 Sprague–Dawley (SD) rats were purchased from Charles River Laboratories (Kingston, NY) and allowed ad libitum access to food and water. All dams were randomly assigned to either Sham-Control (n = 4) or Exposed (n = 5) groups.

**Figure 8:**
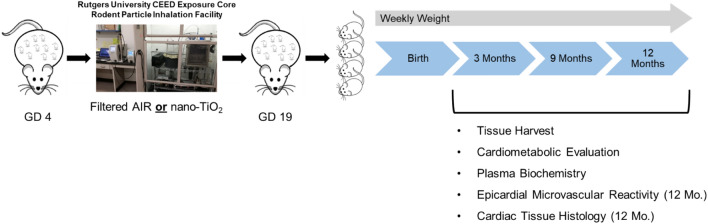
Experimental design. Timed-pregnant Sprague–Dawley rats were assigned to Sham-Control or ENM groups. All animals underwent chronic (4 h/day) whole body exposure to nano-sized titanium dioxide (nano-TiO_2_) aerosols or filtered air, beginning at gestational day (GD) 4. Rats delivered (GD 20–22) and offspring were weighted weekly. Body weight intervals were calculated from the weekly weights based on critical stages of rodent growth. Tissue from progeny was collected at 3 months, 9 months and 12 months for analysis. GD, Gestational day; CEED, Center for Environmental Exposures and Disease.

Rats were exposed for 4 h per day, 5 days per week, during GD 4–GD 19 of pregnancy. This equated to 10.0 ± 0.58 days of exposure. The last exposure was completed approximately 36–48 h before delivery (Fig. 8). This exposure paradigm has previously demonstrated fetal growth restriction and impaired fetal coronary health after gestational exposure^22,24,36,38,39^. For this study we elected to leave dams and litters untouched for one week to prevent maternal rejection of the fetus.

Male and female offspring remained with mothers until weaning, between 3–4 weeks of age, and were randomly assigned to 3, 9, or 12 month groups (n = 8–10 progeny per group). Rat ages extrapolate to approximately 10, 24, and 30 human years, respectively^40^. Care was taken to select one male and one female offspring from each litter for assessment. Over the course of the study, 3 control and 2 exposed offspring were removed from study due to health concerns (Table 1). Animals were housed in an AAALAC accredited facility at Rutgers University. All procedures were approved by the Institutional Animal Care and Use Committee of Rutgers University, were executed in accordance with the standards set forth in the “Guide for the Care and Use of Laboratory Animals” of the National Research Council of the National Academies and was conducted in accordance with ARRIVE guidelines.

### Engineered nanomaterial whole-body inhalation exposure

Maternal exposures to nano-TiO_2_ were performed using a custom rodent inhalation facility designed for whole-body aerosolized nanomaterial inhalation as previously described^37^ (IEStechno, Morgantown, WV). The collective exposure system consists of a vibrating fluidized bed, a Venturi vacuum pump, cyclone separator, impactor and mixing device, an animal housing chamber, and real-time monitoring devices with feedback control. Nano-TiO_2_ aerosols were generated via a high velocity air stream passing through the vibrating fluidized bed and into the Venturi vacuum pump. From there, the aerosols entered the cyclone separator to remove agglomerates greater than 400 nm at an input flow rate of 60 l/min of clean dry air before entering the exposure chamber. Relative mass concentration (9.57 ± 0.3 mg/m^3^; IEStechno, Morgantown, WV) of the aerosols and size distribution (133.73 ± 1.87 nm; SMPS, TSI, Shoreview, MN) and were monitored in real time (Figure S1). Particle concentration was verified through gravimetric sampling wherein aerosols were collected on a 47 nm PTFE membrane filter and an XP2U microbalance (Mettler Toledo, Switzerland).

Each exposure lasted approximately 4 h/day, with a calculated lung daily deposition of 43.8 ± 1.2 μg. Lung deposition was calculated based on previously described mouse methodology and normalized to rat weight and to pregnant rat minute ventilation using the equation: D = F·V·C·T, where F is the deposition fraction (14%), V is the minute ventilation based on body weight, C equals the mass concentration (mg/m^3^), and T equals the exposure duration (minutes)^14,24^. Control animals were exposed within the same inhalation facility to HEPA-filtered air only for 10 days using an identical protocol.

### Body weights

Body weights for all progeny were measured and recorded once a week from the first week after birth to approximately 1 year.

Growth data was analyzed in accordance with an approach to the evaluation of rodent growth data in toxicology studies by Hoffman et al.^41^. Weekly data were reported for the first four weeks. Growth data from subsequent weeks were pooled across relevant time intervals to obtain an interval average for each animal within each analysis interval. After collecting weekly data for the first 4 weeks after birth, a three-week moving average was calculated every two weeks at weeks 5, 7, 9, 11, and 13. Thereafter, a five-week moving average was calculated every 4 weeks at weeks 16, 20, and 24 followed by a fifteen-week moving average every 14 weeks at weeks 33, and 47. Data is reported as male, female, and combined progeny for both the control and exposed groups.

### Mean arterial pressure (MAP)

Rats were anesthetized with isoflurane gas (5% induction, 3% maintenance). The right carotid artery was cannulated to directly assess mean arterial pressure (MAP). A BLPR2 pressure transducer (World Precision Instruments, Sarasota, FL) was used in conjunction with a blood pressure monitor (World Precision Instruments, Sarasota, FL) to measure MAP.

### Isolated microvessel preparation

Isolated microvessel responses were recorded in a subset of animals at 12 months of age. Following blood collection, the heart was removed, flushed of excess blood, and placed in physiological salt solution (PSS (in mmol/l): 129.8 NaCl, 5.4 KCl, 0.5 NaH_2_PO_4_, 0.83 MgSO_4_, 19 NaHCO_3_, 1.8 CaCl_2_, and 5.5 glucose; pH 7.35–7.40) and chilled to 4 °C for dissection. Coronary resistance arterioles (< 160 um maximum diameter) from the left anterior descending (LAD) artery distribution were isolated, excised, and transferred to a vessel chamber (Living Systems Instrumentation, Burlington, VT, USA) containing oxygenated PSS as previously described^28,42^. Vessel segments were cannulated with glass pipettes and secured using nylon suture (11–0 ophthalmic, Alcon, UK). Arterioles were extended to their in situ length, pressurized to 45 mmHg with PSS, superfused with warmed (37 °C) oxygenated (21% O_2_–5% CO_2_–74% N_2_) PSS at a rate of 10 ml/minute and allowed to develop spontaneous tone over 30 min of equilibration. Vessel diameters were measured using video calipers (Colorado Video, Boulder, CO, USA).

Following equilibration arteriolar responsiveness to chemical agents was assessed randomly to avoid any ordering effects. Relaxation responses were evaluated via cumulative addition of 100 µL acetylcholine (ACH; 1 × 10^−9^ to 1 × 10^−4^ M; MP Biomedicals LLC, Santa Ana, CA, USA) to assess endothelium-dependent reactivity or sodium nitroprusside (SNP; 1 × 10^−9^ to 1 × 10^−4^ M; Thermo Fisher Scientific, Waltham, MA, USA) to assess endothelium-independent reactivity. Following assessments of arteriolar reactivity, the superfusate was replaced with Ca^2+^-free PSS to establish passive tone. All pharmacological agents were dissolved in PSS.

Spontaneous tone was calculated by the following equation: [(D_M_ − D_I_)/D_M_] × 100, where D_M_ is the maximal diameter recorded at 45 mm Hg for the coronary arterioles under Ca^2+^-free PSS, and D_I_ is the initial steady-state diameter achieved prior to the experimental period.

The responses to ACH and SNP are presented as percent relaxation from spontaneous baseline diameter: [(D_SS_ − D_CON_)/(D_M_ − D_CON_)] × 100, where D_SS_ remains the steady-state diameter achieved after each chemical bolus, and D_CON_ is the control diameter measured immediately prior to the dose–response experiment. All experimental periods were at least two minutes, and all steady-state diameters were collected for at least one minute. These studies were not blinded as the researcher performing these assessments (PS) also prepared the animals for exposure.

### Histology

All histological examinations were performed using standard laboratory procedures.

Representative hearts from a subset of animals at 12 months of age were fixed in 10% neutral buffered formalin, embedded in paraffin blocks, and sectioned to 4 µm-thick sections that were subsequently mounted onto glass slides. Hematoxylin and eosin (H&E) stained slides were assessed by an ACVP board-certified veterinary pathologist who was blinded to the treatment group.

### Blood collection

Under isoflurane sedation, blood was collected for clinical chemistry prior to euthanasia. Approximately 4–5 cc of blood was collected directly from the carotid artery cannula and divided into EDTA vacutainers. Blood was centrifuged (1100 RCF, 10 min) to separate the plasma from other constituents. After centrifugation plasma was removed with a transfer pipette, flash frozen in liquid nitrogen, and stored at − 80 °C until analysis. Hearts were removed at sacrifice, after rinsing with 4 °C physiological salt solution, hearts were blotted on a Kimwipe® and weighed.

### Plasma biochemistry

Plasma total cholesterol (TC, Pointe Scientific, Canton, MI), auto low density lipoprotein (autoLDL, Pointe Scientific, Canton, MI), auto high density lipoprotein (autoHDL, Pointe Scientific, Canton, MI), and triglyceride (TG, Pointe Scientific, Canton, MI), concentrations were quantitated in duplicate using enzymatic assays with commercially available reagents (Pointe Scientific, Canton, MI) according to manufacturer’s instructions in progeny at 3, 9 and 12 months of age.

### Multiplex protein analysis

Circulating concentrations for cytokines and chemokines (Leptin, TNFɑ, GRO IL-4, IL-1β, IL-6, IL-10, IFNγ, MCP-1, VEGF, RANTES) in plasma from Control and Exposed animals were estimated using a Millipore Rat Cytokine/Chemokine Assay (EMD Millipore) at 3, 9, and 12 months of age. Briefly, specific proteins bound to fluorescent beads were detected using a mix of protein-specific, biotinylated detector antibodies followed by incubation with streptavidin-PE. Circulating concentrations for biomarkers of vascular injury (sICAM-1 and sE-Selectin) in plasma from Control and Exposed animals were estimated using a Millipore Rat Vascular Injury Assay (EMD Millipore).

### Statistics

All data, with the exception of growth, were assessed by maternal dam not individual progeny. Growth data was analyzed between treatment groups using a two-way ANOVA and was followed by the least-significant difference test (LSD) for multiple comparisons. All statistical analysis was completed with GraphPad Prism 8 (San Diego, CA, USA). Point-to-point differences in the body weight were evaluated using two-way repeated measures analysis of variance (ANOVA) followed by LSD post hoc multiple comparison. Metabolic, inflammatory, and vascular injury data were evaluated using two-way repeated measures analysis of variance (ANOVA) with a Tukey’s post hoc analysis when significance was found. Pressure myography data was evaluated using first-order regression equations developed to assess line slope relationships (SigmaPlot 11.0, Systat, San Jose, CA). All data are expressed as mean ± SEM and significant results are indicated at *p* ≤ 0.05.

## Supplementary Information

Supplementary Information.
