# Supplementary material for: Development of coronary dysfunction in adult progeny after maternal engineered nanomaterial inhalation during gestation
Source: Sci Rep. 2021 Sep 29;11:19374. doi: 10.1038/s41598-021-98818-8 (PMC8481306; doi:10.1038/s41598-021-98818-8)
Supplement: Supplementary file 1 — Supplementary Information. [file 41598_2021_98818_MOESM1_ESM.docx]

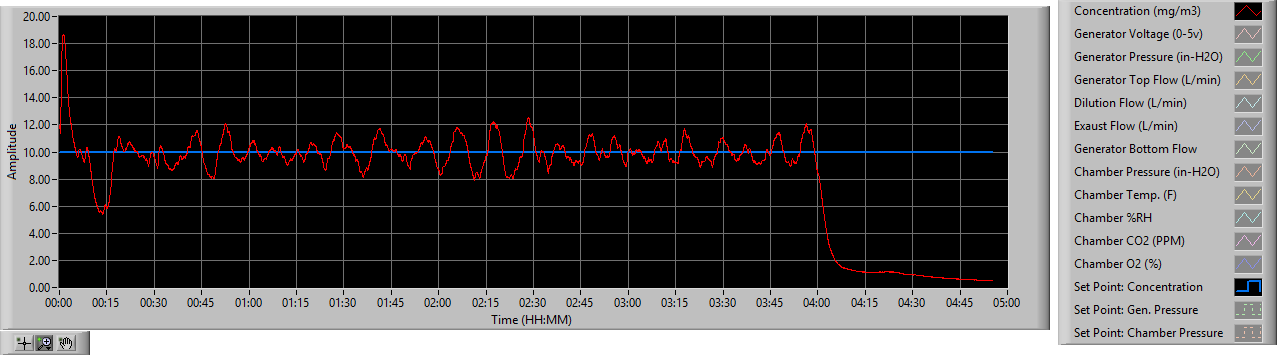


**Supplementary Figure 1. ENM aerosol generation and characterization.** Characterization of nano-TiO_2_. (A) Real-time mass concentration measurements of nano-TiO_2_ aerosol during a representative inhalation exposure. The blue line represents the target concentration, 10 mg/m^3^. The red line represents the real time measurement of aerosol concentrations within the inhalation chamber, in this representative example the concentration was 9.57 ± 0.3 mg/m^3^. (B) Size distribution of nano-TiO_2_ aerosol (mobility diameter) determined using a scanning mobility particle sizer (SMPS; count median diameter = 125.7 ± 0.38 nm).

Figure (SUPPLEMENTARY 1)
INHALATION EXPOSURE

9.57 ± 0.3 mg/m^3^

A

B

133.73 ± 1.87 nm
